# FtsZ does not initiate membrane constriction at the onset of division

**DOI:** 10.1038/srep33138

**Published:** 2016-09-09

**Authors:** Daniel O. Daley, Ulf Skoglund, Bill Söderström

**Affiliations:** 1 Center for Biomembrane Research Department of Biochemistry and Biophysics Stockholm University, SE-106 91 Stockholm, Sweden; 2 Structural Cellular Biology Unit Okinawa Institute of Science and Technology Okinawa 904-0495, Japan

## Abstract

The source of constriction required for division of a bacterial cell remains enigmatic. FtsZ is widely believed to be a key player, because *in vitro* experiments indicate that it can deform liposomes when membrane tethered. However *in vivo* evidence for such a role has remained elusive as it has been challenging to distinguish the contribution of FtsZ from that of peptidoglycan-ingrowth. To differentiate between these two possibilities we studied the early stages of division in *Escherichia coli*, when FtsZ is present at the division site but peptidoglycan synthesizing enzymes such as FtsI and FtsN are not. Our approach was to use correlative cryo-fluorescence and cryo-electron microscopy (cryo-CLEM) to monitor the localization of fluorescently labeled FtsZ, FtsI or FtsN correlated with the septal ultra-structural geometry in the same cell. We noted that the presence of FtsZ at the division septum is not sufficient to deform membranes. This observation suggests that, although FtsZ can provide a constrictive force, the force is not substantial at the onset of division. Conversely, the presence of FtsN always correlated with membrane invagination, indicating that allosteric activation of peptidoglycan ingrowth is the trigger for constriction of the cell envelope during cell division in *E. coli*.

In most bacteria a large, highly dynamic protein complex called the ‘divisome’ assembles at the midcell, constricts the cell envelope and finally separates the mother cell into two daughter cells^1^,^2^,^3^,^4^. Despite considerable effort, it is not completely clear which proteins in the divisome that trigger membrane invagination, or contribute to constriction of the cell envelope.

At the center of the divisome complex is FtsZ, a protein that forms a structural framework for the rest of the divisome^5^. Numerous *in vitro* studies posit that FtsZ is a major force generator during constriction^6^,^7^,^8^,^9^. Perhaps the most convincing observation backing this postulate is that, when artificially fused to the membrane, FtsZ exerts a contractile force on liposomes^7^,^8^,^10^. Whether this contractile force is significant *in vivo* remains an important but unanswered aspect of cell division. A secondary source for envelope constriction most likely comes from peptidoglycan (PG) ingrowth. PG forms a rigid layer that needs to be remodeled at the division site; this process is carried out by amidases (AmiA, AmiB, AmiC) and their respective activators (EnvZ, NlpD), transglycosylases (PBP1A, PBP1B, MgtA) which together with FtsW forms the septal PG synthase complex, the sensory subcomplex FtsQ/L/B, a transpeptidase (FtsI) and its allosteric activator (FtsN)^11^,^12^,^13^,^14^,^15^. Perturbations to many of these PG synthesising/regulating enzymes inhibit division and result in filamentous cells. These filaments often contain multiple FtsZ-rings, that appear not to constrict, as judged by the relatively crude resolution obtainable by fluorescence microscopy^16^,^17^,^18^,^19^,^20^. However, it is not possible to determine if geometrical changes take place at a membrane level using fluorescence microscopy alone.

In order to determine whether a divisome protein is contributing to constriction of the envelope *in vivo*, it is essential to be able to monitor its localization at the divisome, and at the same time the ultra-structure of the membranes (to detect small invaginations). To achieve this we have, in this study, used correlative cryo-fluorescence and cryo-electron microscopy (cryo-CLEM) as it is currently the only method that enables tracking of fluorescently labeled proteins *in vivo* and high-resolution cryo-electron microscopy (cryo-EM) imaging of membrane ultra-structure in the same cell^21^,^22^ (Supplementary Fig. 1 for an overview of the workflow). Using this approach we have monitored the contributions of FtsZ, FtsI and FtsN during the initial stage(s) of envelope constriction.

## Results

### FtsZ does not generate a strong enough force to deform the inner membrane

**Figure 1:**
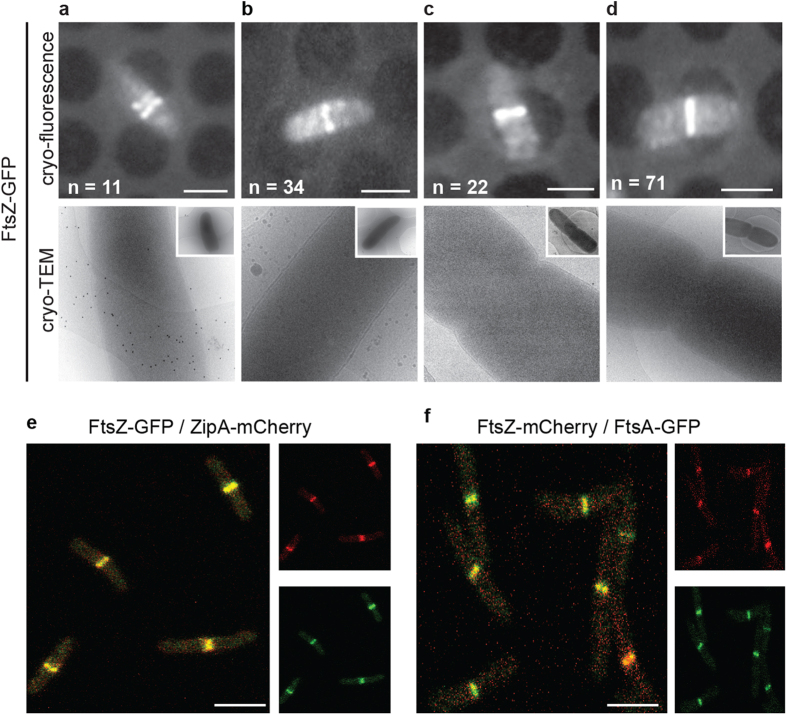
The presence of FtsZ at the division septum is not enough to initiate inner membrane constriction. *E. coli* cells expressing chromosomally encoded FtsZ-GFP were analysed by cryo-CLEM. (**a**–**d**) Upper row, cryo-fluorescence image. Lower row, cryo-electron microscopy image of the same cells as above. (**a**) In cells where FtsZ-GFP had not yet condensed to a single ring, membrane invagination was not initiated. (**b**) Cells with FtsZ-GFP accumulated at the midcell, but without visible constrictions. (**c**,**d**) Cells with FtsZ-GFP accumulated at the midcell that also showed visible constrictions, indicative of a later stage during division. All cells in this stage had uniform inner and outer membrane invaginations. FtsZ-GFP was also observed in deeply constricted cells (Supplementary Fig. 3), but was not observed in cells that had completed division. Cells expressing FtsZ-GFP exhibited no apparent growth phenotype (Supplementary Figs 4 and 6) and the amount of FtsZ-GFP was less than 20% of the total cellular FtsZ (Supplementary Fig. 5). The total number cells examined by cryo-CLEM during early FtsZ-GFP accumulation at midcell was 127 (the total number of cells for all stages was >200). (**e**,**f**) The membrane tethers FtsA and ZipA localize to midcell together with FtsZ, shown by dual color fluorescence microscopy imaging on live cells simultaneously expressing (**e**) FtsZ-GFP and ZipA-mCherry or (**f**) FtsZ-mCherry and FtsA-GFP. *n *> 100. Scale bars = 2 μm. Images are best viewed on a digital screen.

Our initial goal was to determine if FtsZ was sufficient to generate a contractile force *in vivo.* Our working hypothesis was that, if FtsZ was sufficient we should see deformations of the inner membrane when only FtsZ and its membrane tethers FtsA^23^ and ZipA^24^ (aka the proto-ring) are present at midcell (but PG synthesizing enzymes are not). We cryogenically preserved *Escherichia coli* cells expressing a chromosomal copy of FtsZ-GFP by vitrification and imaged them by cryo-CLEM (Supplementary Fig. 2). When examining cells in a pre-divisional stage, indicated by the typical helical arrangement of FtsZ-GFP^25^,^26^, we never observed membrane invagination (Fig. 1a). In cells where FtsZ-GFP had condensed to a single band (as judged from the cryo-fluorescence images) we noted that 27% lacked a visible invagination (as judged from the cryo-EM images) (Fig. 1b). The cells that had visible membrane invaginations, could be further classified as having either minor (~17%) (Fig. 1c) or major invaginations (~56%) (Fig. 1d). In all cells where membrane invagination was observed we noted that both the inner and outer membranes were equally deformed (see also Supplementary Fig. 3). These observations suggest that the presence of FtsZ-GFP at the division septum is not sufficient to deform the inner membrane *in vivo*.

### The proto-ring assembles at one point in time

The inability of FtsZ to deform the inner membrane *in vivo* could be explained by the possibility that it was arriving at the midcell before ZipA and FtsA, and was therefore not yet tethered to the inner membrane. Although previous studies have shown FtsZ, ZipA and FtsA arrive at the division site at essentially the same point in time^23^,^27^, we validated this point using our experimental setup. We co-expressed FtsZ-GFP and ZipA-mCherry and monitored their localization at the mid-cell by live cell dual color fluorescence microscopy imaging (Fig. 1e). In all cells analyzed (n > 100), both fusions co-localized at the mid-cell. Or stated in an alternative way, there was no instance where FtsZ-GFP was present at the midcell and ZipA-mCherry was not. The same observation was made when we co-expressed a plasmid-encoded version of FtsZ-mCherry and a chromosomally encoded version of FtsA-GFP (Fig. 1f). As a result we can conclude that the whole proto-ring is assembled in our cryo-CLEM experiments on FtsZ-GFP. The most plausible explanation for our earlier observations using cryo-CLEM is that FtsZ was tethered by ZipA and FtsA, but that it was simply not generating a sufficient force to deform the inner membrane.

### Cephalexin treated cells does not show membrane invagination at potential division sites

Additional evidence that the proto-ring containing FtsZ, FtsA and ZipA is not generating a sufficient force to deform the inner membrane was obtained by adding the antibiotic Cephalexin (15 μg/ml) to cultures of cells expressing FtsZ-GFP and analyzing potential division sites by cryo-CLEM (Supplementary Fig. 7). Cephalexin allows the early division proteins to assemble, but inhibits the catalytic activity of a down stream protein (FtsI) which in turn blocks cell division and renders cells filamentous^28^,^29^. We reasoned that if FtsZ would exert a large enough force to constrict membranes *in vivo* we would detect membrane invaginations in the filaments at potential division sites using cryo-CLEM. Cephalexin treated cells did not contain membrane invaginations at spots where FtsZ-GFP was accumulated (n = 27) (Supplementary Fig. 7). These data taken together with the previous cryo-CLEM data strongly support a scenario in which FtsZ-GFP does not generate a large enough force to invaginate membranes *in vivo*.

Significantly, immunofluorescence microscopy has shown that FtsZ-GFP mirrors the localization patterns of its native counterpart, thus we can be confident that also the native FtsZ was present in cells lacking visible invaginations^30^,^31^,^32^.

### FtsN accumulation at midcell triggers membrane invagination

If FtsZ is not sufficient to initiate the membrane invagination, what is? The other plausible candidate is peptidoglycan (PG) ingrowth^1^, a process largely governed by two divisome proteins that arrive at a later time point than FtsZ, FtsI and FtsN^33^. FtsI is a PG synthase that is thought to be allosterically activated by FtsN^12^. FtsN is the last essential protein to arrive at midcell and is generally believed to be responsible for the initiation of membrane invagination^12^, however it has not been possible to unequivocally resolve this point because available methods such as super resolution fluorescence microscopy were unable to correlate the presence of FtsN with membrane invagination. Moreover recent data has suggested that a portion of the septal localized FtsN is recruited early by FtsA^34^, and that this portion of FtsN is independent of FtsI localization to midcell^35^. These observations raised the possibility that membrane invagination could be initiated earlier than previously thought.

**Figure 2:**
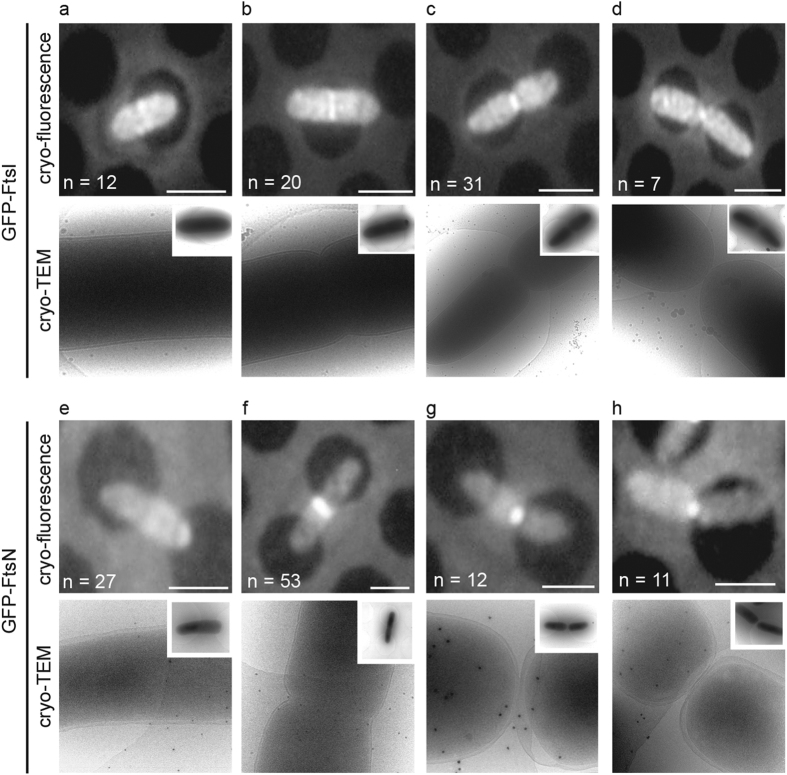
The arrival of FtsN coincides with the onset of membrane constriction at the division septum. *E. coli* cells expressing a chromosomal copy of either GFP-FtsI or GFP-FtsN were subjected to cryo-CLEM. Upper row, cryo-fluorescence image. Lower row, cryo-electron microscopy image of the same cells as above. (**a**–**d**) Pseudo time-lapse images of cells expressing GFP-FtsI. (**a**) GFP-FtsI is present at the midcell prior to the onset of membrane invagination. (**b**–**d**) It then remains at the septum throughout membrane constriction and persists until the cell envelope closes. (**e**–**h**) Pseudo time-lapse images of cells expressing GFP-FtsN. (**e**) Cells expressing GFP-FtsN without a clear accumulation of a fluorescent signal at the midcell show no visible membrane invagination. (**f**) Upon the arrival of GFP-FtsN at the midcell, the membranes begin to constrict. (**g**,**h**) GFP-FtsN is localized at the division septum throughout membrane constriction and remains until full closure of the cell envelope. Insets show whole cells for orientation. Scale bars = 2 μm. At least 70 cells of each strain were analysed by cryo-CLEM (*n* = number of cells in each representative stage). Images are best viewed on a digital screen. Black dots in the cryo-TEM images are 20 nm gold beads added for size reference.

To determine if the accumulation of FtsI or FtsN to midcell was sufficient to initiate constriction of the inner membrane *in vivo* we expressed either a chromosomal encoded GFP-FtsI or GFP-FtsN and analyzed cells by cryo-CLEM. Here we present pseudo time-lapse images of respective protein during different stages of division (Fig. 2a–h). In cells where GFP-FtsI was accumulated at the septum, we observed that roughly 35% were without a visible membrane invagination at the midcell (Fig. 2a). Thus accumulation of FtsI at the septum is clearly not enough to initiate membrane invagination. The remaining 65% of cells showed both GFP-FtsI accumulation and membrane invagination (Fig. 2b). These numbers were comparable to what we observed for FtsZ-GFP, suggesting that FtsI is not sufficient to initiate constriction of the membranes.

Conversely, in cells where GFP-FtsN was accumulated at midcell we observed that 100% of cells (*n* = 65) contained visible membrane invaginations (Fig. 2f,g). These invaginations again indicated a uniform constriction of both the inner and outer membranes and could either be minor (Fig. 2f) or major (Fig. 2g). Even though we cannot formally rule out the possibility that membrane invagination is initiated prior to FtsN arrival at the septum, the data presented here strongly suggests that FtsN accumulation at midcell is a trigger for membrane invagination. Finally, we also noted that both GFP-FtsI and GFP-FtsN remained at the septum until complete closure of the cell envelope (Fig. 2d,h).

## Discussion

In summary, we have utilized cryo-CLEM to study the early stages of bacterial cell division. This novel approach allowed us to correlate the localization of specific proteins at the division septum with the underlying membrane geometry. The latter allowed us to go far beyond the current resolution limit of light microscopy and observe minor membrane invaginations that mark the onset of envelope constriction. Our data unequivocally demonstrate that the presence of FtsZ (together with ZipA and FtsA) at midcell, is not sufficient to initiate membrane constriction *in vitro*. This conclusion does not contradict previous studies that show that membrane anchored FtsZ polymers can generate a force on membranes *in vitro*^7^,^8^,^36^. But it does suggest that the force generated is not strong enough to initiate membrane invagination *in vivo*. Taking into account other recent findings, which show that (1) FtsZ activity is not a rate-limiting factor during membrane constriction^30^, (2) that it is not present at a late stage of division^31^, and (3) the fact that cell wall free *E. coli* can undergo division in absence of FtsZ^37^, brings its role as a main force generator into question. Nonetheless it is still possible (even probable) that it contributes to constriction.

In the course of our experimentation we also observed that the assembly of FtsI was not sufficient to initiate constriction. However, once its allosteric activator FtsN had assembled we observed that all cells had visible invaginations. The simplest interpretation of this latter observation is that FtsN acts as the trigger for envelope constriction, by activating FtsI, other sensory proteins (FtsQ/B/L) and PG in-growth. Although this claim has been made previously^14^,^15^,^28^, it was based on low-resolution fluorescence imaging, which cannot resolve the ultra-structure of the membrane. Our cryo-CLEM data are therefore the first to emphatically prove this point.

## Methods

### Cryo-CLEM workflow

The workflow of Koning *et al*.^38^ was followed loosely. Each individual step is detailed below and schematically depicted in Supplementary Fig. 1. Briefly, cells were grown and the respective fluorescent protein fusion was produced by the addition of Isopropyl β-D-1-thiogalactopyranoside (IPTG), thereafter an aliquot was transferred to a Zeiss Elyra SP.1. for a pre-plunging fluorescence check using Structured Illumination Microscopy (SIM). Following SIM imaging, another aliquot from the same culture was mixed (10:1) with 20 nm gold beads, and 1–5 μl sample was directly applied on glow discharged EM girds and plunge frozen in liquid Ethane using a Vitrobot (FEI company, The Netherlands). Cryo-fluorescence images were acquired using a Linkam cryo-stage (Linkam Scientific, UK) mounted on an upright Zeiss Axio Imager A2 microscope, and cells with different fluorescence localization were identified. The grids were subsequently transferred to a Talos ARCTICA cryo-TEM (FEI Company, The Netherlands), where the same cells again were identified in low magnification mode before high magnification imaging was preformed. Data correlation was finally performed using ImageJ and Adobe Photoshop. Note that EM grids are round and may rotate during sample transfer, thus cryo-fluorescence and cryo-TEM images may be at slightly different angles with respect to each other.

### Strains

Construction of strains BS001, BS003 and BS007 carrying chromosomal encoded copies of FtsZ-GFP, FtsA-GFP and GFP-FtsI respectively, is described elsewhere^31^. Strain EC1213 carrying a chromosomal encoded copy of GFP-FtsN (MG1655 attHK22::pDSW560 (lacIq, P207::gfp-ftsN))^32^ was a gift from David Weiss (University of Iowa). All strains and plasmids used in this study can be found in Supplementary Table 1.

### Growth conditions

Cells were grown (37 °C) in LB overnight and back-diluted 1:200 in fresh media and grown for 1 hour before inducing production of fluorescent proteins. Antibiotics were added at the appropriate concentration (Ampicillin 25 μg ml^−1^, Kanamycin 50 μg ml^−1^, Cephalexin 15 μm ml^−1^). Cells in early exponential growth phase were harvested and imaged by SIM and cryo-CLEM.

### Fluorescent protein production

As described before^31^,^32^, production of FtsZ-GFP, FtsA-GFP, GFP-FtsI and GFP-FtsN was induced with Isopropyl β-D-1-thiogalactopyranoside (IPTG) at the following concentrations; FtsZ-GFP, 2.5 μM; FtsA-GFP, 100 μM; GFP-FtsI and GFP-FtsN, 5 μM. FtsZ-mCherry was produced from pEG4^39^ and ZipA-mCherry^32^, as described.

### Structured Illumination Microscopy (SIM) Imaging

~6 μl of cell culture was placed on a microscope glass coated with thin pre-made agarose pads (1% (w/v) agarose). A cover slip was added and the cells were left for ~5 minutes so that they had sufficient time to immobilise. SIM images were acquired on a Zeiss ELYRA PS.1 system using a 100X NA 1.4 oil immersion plan-Apochromat objective. GFP was excited at a wavelength of 488 nm and mCherry at 561 nm. To minimize bleaching and photo damage to the cells during imaging, the laser power output was kept below 2,5% for the 488 nm laser, and 5% for the 561 nm laser (total laser power was 5 and 10 mW respectively). The calibrated SIM system gave a lateral (xy-) resolution of ~100 nm. For each fluorophore the illumination grids were phase shifted 3 times over the field of view (1024  ×  1024) and then rotated 180/3 degrees (i.e. three rotations per image) to generate a data set containing 9 raw images per fluorophore. Appropriate laser and filter settings for the respective fluorophore were chosen in the software ZEN2011 Black. The SIM images were reconstructed and subsequently analysed in ZEN2011 Black or ImageJ (N.I.H.). Imaging was preformed at controlled room temperature of 23.5 °C.

### Vitrification

Quantifoil grids (R3,5/1 or R2/2 Cu 200 mesh) were glow discharged and 3–5 μl of sample solution was directly applied. Grids were blotted using Whatman filter papers and plunge frozen using a Vitrobot (FEI Company, The Netherlands) in pre-cooled liquid Ethane. The samples were subsequently stored under liquid nitrogen conditions until further examination.

### Cryo-Fluorescence Microscopy imaging

A Linkam CMS196 cryo-stage was mounted on an upright Zeiss Axio Imager A2 microscope body equipped with an Olympus DP73 Peltier cooled CCD camera, a (cryo compatible) Zeiss air LD EC Epiplan-NEUFLUAR 100 × 0,75 NA objective with a working distance of 4.0 mm and an ‘GFP’ filter cube (Ex. 470/40. FT 495. Em. 525/50). Images were recorded using Olympus cellSens software (Olympus, Japan). Once a grid was examined it was directly removed form the cryo-stage, mounted into a FEI autogrid and again stored in liquid nitrogen until further examination.

### Cryo-Transmission Electron Microscopy imaging

Grids that already had been examined by cryo-fluorescence imaging and found to contain interesting regions of interests were transferred to a Talos ARCTICA 200 keV cryo-transmission electron microscope equipped with a Falcon II direct electron detector. The regions of interests identified in the cryo-fluorescence step were manually relocated using low magnification mode (84×–210×). Once the same area as in the fluorescence images (*i.e.* the cells) had been localized, high magnification (4300×–57000×) imaging was preformed using low dose mode.

### Image post processing

Noise reduction of the cryo-electron microscopy data, was preformed using the COMET^40^ software operated in 2D mode. Fluorescence images were brightness adjusted and cropped using ImageJ (N.I.H, USA). Finally, all images were transferred to Abode Illustrator for figure preparation.

## Additional Information

**How to cite this article**: Daley, D. O. *et al*. FtsZ does not initiate membrane constriction at the onset of division. *Sci. Rep.* **6**, 33138; doi: 10.1038/srep33138 (2016).

## Supplementary Material

Supplementary Information
