# Supplementary material for: FtsZ does not initiate membrane constriction at the onset of division
Source: Sci Rep. 2016 Sep 9;6:33138. doi: 10.1038/srep33138 (PMC5016829; doi:10.1038/srep33138)
Supplement: Supplementary Information [file srep33138-s1.pdf]

Supporting material for:

FtsZ does not initiate membrane constriction at the onset of division

Daniel O. Daley[a], Ulf Skoglund[b] and Bill Söderström[b]

[a]Center for Biomembrane Research
Department of Biochemistry and Biophysics
Stockholm University, SE-106 91 Stockholm, Sweden
[b]Structural Cellular Biology Unit
Okinawa Institute of Science and Technology
Okinawa 904-0495, Japan

Supplementary Figure 1

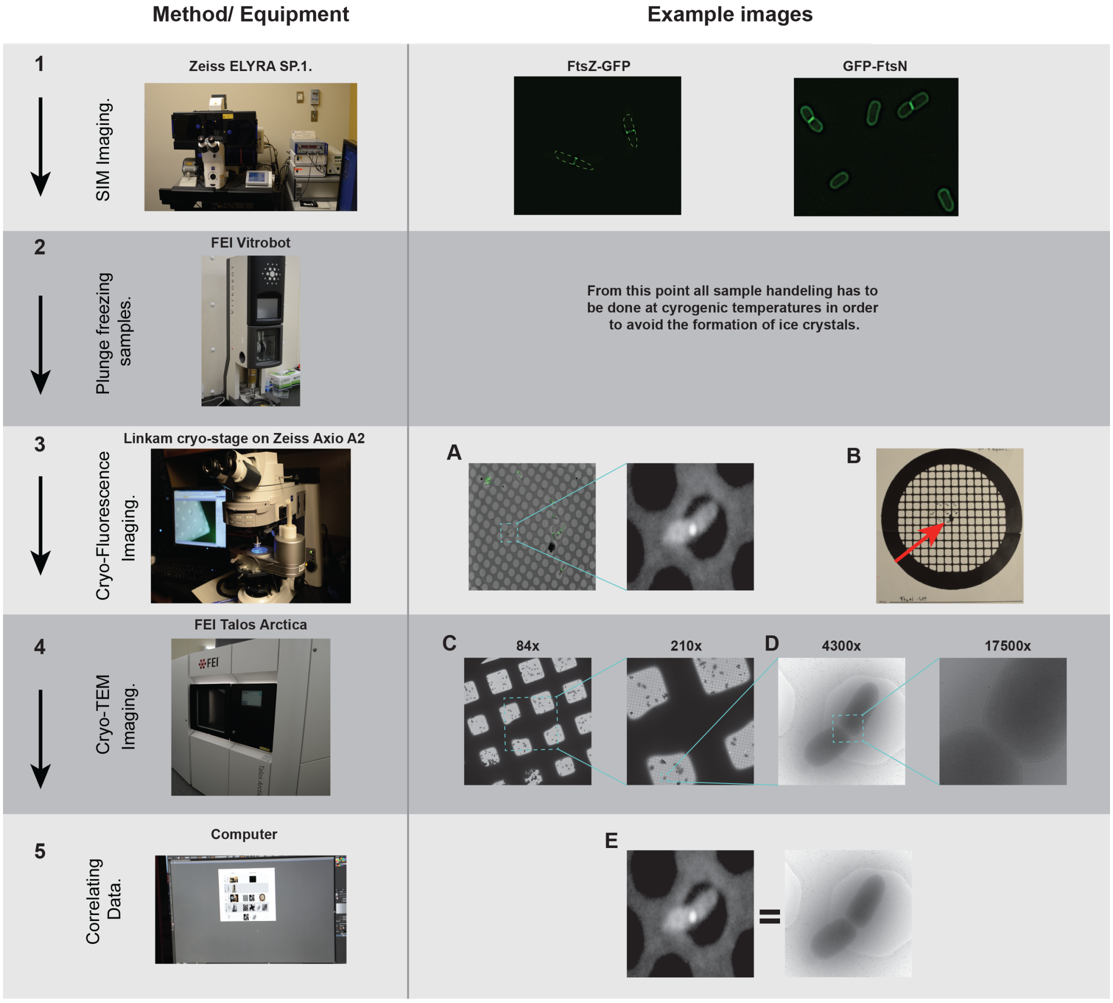

Schematic overview of the cryo-CLEM workflow.

1. Fluorescence (SIM) imaging.

   Performed prior to plunge freezing in order to ensure that cells were expressing the fluorescent protein fusion at an appropriate level. Representative images of FtsZ-GFP and GFP-FtsN are shown.

2. Plunge freezing of samples.

   3-5 µl from the same culture was then applied on glow discharged Quantifoil R3.5/1 or R2/2 200 mesh Cu grids and plunge frozen in liquid

ethane. Plunge freezing of samples in liquid ethane ensures the preservation at a nearly native state of the cells[1]. From this point the samples are maintained at a liquid nitrogen environment to prevent formation of ice crystals.

3. Cryo-fluorescence microscopy.

   Performed using a Linkam cryo-stage mounted on an upright Zeiss Axio Imager.A2 microscope. Interesting cells were identified and imaged (**3A**), and their position on the EM grid was manually recorded (**3B**) for later correlation with the EM data.

4. Cryo-Electron microscopy.

   Samples were transferred to a cryo-TEM for EM data collection. Same region of interest was identified using the notes from (**3B**). Low magnification (84x-210x) images (**4C**) were acquired for overview orientation and subsequently high magnification (4300x -57000x) images were acquired (**4D**) and stored for later correlation. A few cells were also subjected to cryo-ET.

5. Data correlation.

   In a final step the cryo-fluorescence and cryo-TEM data were correlated and the same cells from both data sets were identified (**5E**) and subsequently analyzed.

Although it is a powerful technique, there are limitations to this cryo-CLEM approach. One drawback is the limitation in the spatial resolution. Due to the relatively low NA (0.75) of the objective we used while acquiring the cryo-fluorescence images the resolution will be limited to a few hundred nanometers (comparable to normal wide field fluorescence microscopy). There are ways to improve on the spatial resolution of the cryo-light microscope using photoactivatable fluorescent proteins in order to allow a higher accuracy in localizing the fluorescent reporters (*i.e.* cryo-PALM[2]). However, the photoswitching capability of fluorescent proteins is heavily dampened in cryo conditions, and the need for high laser power, are two major concerns using that approach. Since we wanted to image a structure of known position we reasoned that sub diffraction localization at the cost of potential cell damage due to high laser power was not warranted. Another limitation of cryo-fluorescence microscopy is that, even though the photobleaching is reduced[3], the overall fluorophore brightness may be dampened if the ice layer is not thin enough. We found that our GFP-fusions performed well as long as the ice thickness was kept at < the cellular thickness.

Supplementary Figure 2

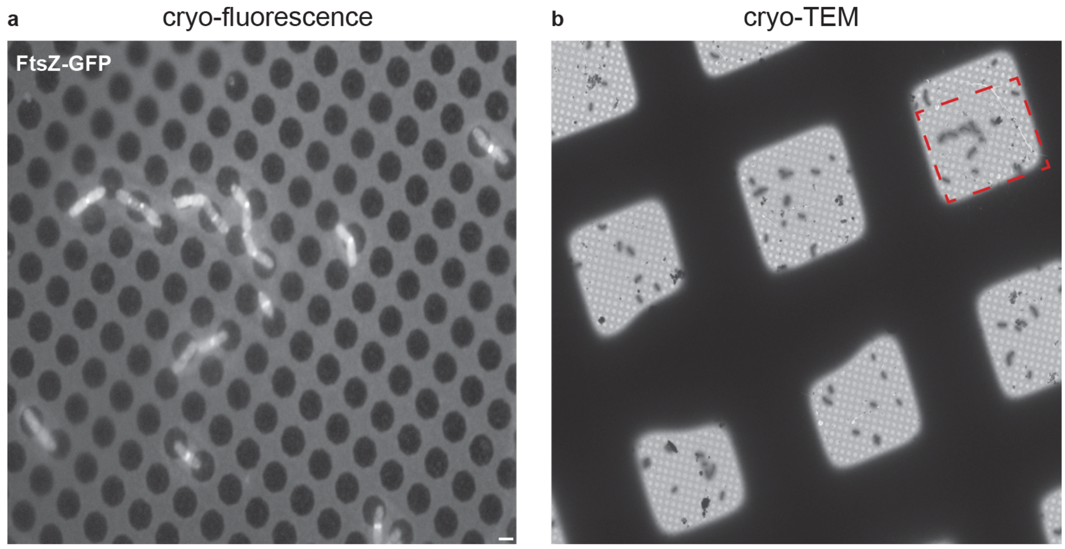

Cryo-fluorescence and cryo-transmission electron microscopy (cryo-TEM) on the same sample. Correlative cryo-CLEM allows sequential image acquisition by cryo-fluorescence then cryo-TEM on the same sample. (**a**) Cryo-fluorescence image of *E. coli* cells expressing chromosomal encoded FtsZ-GFP. Scale bar = 2 μm. (**b**) Cryo-TEM image of the same cells. The red square in (**b**) is roughly the same area as in (**a**).

Supplementary Figure 3

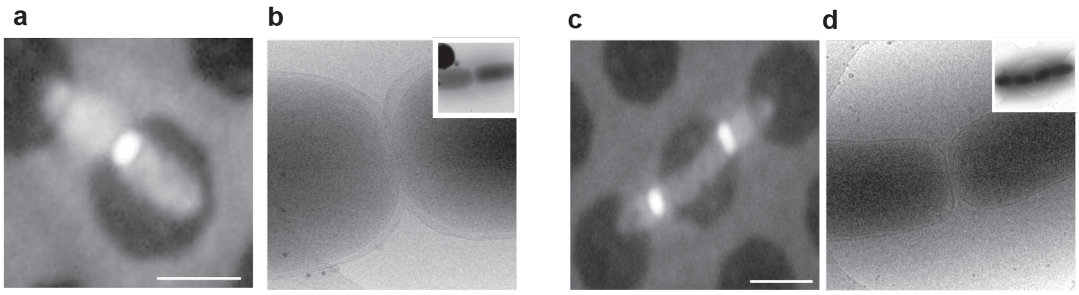

Cryo-CLEM of *E. coli* cells expressing FtsZ-GFP during the later stages of cell division. For comparison we also imaged cells that were in late stages of division. (**a** and **b**) When FtsZ-GFP condensed down to a spot membrane invagination had progressed further. (**c** and **d**) By the time FtsZ-GFP was observed at future division sites in daughter cells, the new poles appeared to have formed. Insets show whole cells for orientation. Scale bars = 2 µm.

Supplementary Figure 4

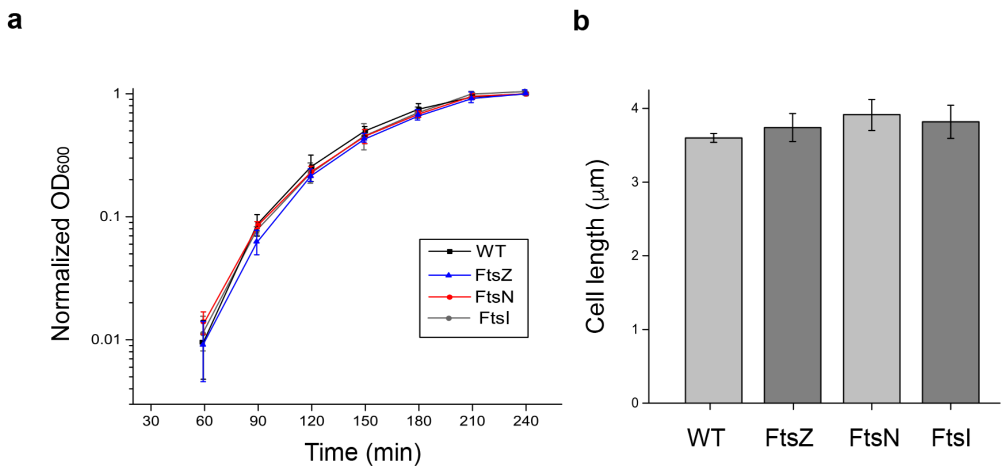

c

Protein	Number of cells with mid cell localization*	% of cells with mid cell localization	% new born cells with mid cell localization	n_{NB}	n_{Tot}
FtsZ	215	93	91	62	231
FtsN	132	68	0#	29	194
FtsI	122	73	0#	17	167

* , Determined by visual inspection of SIM images

\# , New born cells did not have FtsN located at midcell since it arrives late in divisome assembly

n_{NB}, Cells not in a divisional state (New born), n_{Tot}, Total number of cells scored

Cell viability.

Cells expressing fluorescent protein fusions BS001 (FtsZ-GFP), EC1213 (GFP-FtsN) and BS007 (GFP-FtsI) behave as WT cells during growth in liquid culture at 37 ^{o}C. (**a**) Growth curves indicated similar growth rates. *n* = 3. (**b**) Cell length measurements showed that all strains were comparable in length. *n* > 100 for each strain. (**c**) Frequency of rings observed in the cells expressing different fluorescent proteins. These numbers are in line with what has been previously reported [4-7]. Collectively, these experiments indicate that strains producing FtsZ-GFP, GFP-FtsN and GFP-FtsI grew comparably with WT cells.

Supplementary Figure 5

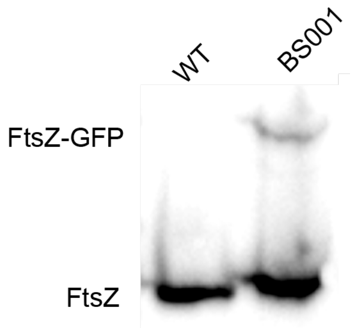

FtsZ-GFP expression levels.

Western blot analysis of the expression level of native FtsZ and FtsZ-GFP. First lane: WT *E. coli* cells. Second lane: BS001 (expressing FtsZ-GFP). FtsZ-GFP accounted for < 20 % of the total amount of cellular FtsZ.

Supplementary Figure 6

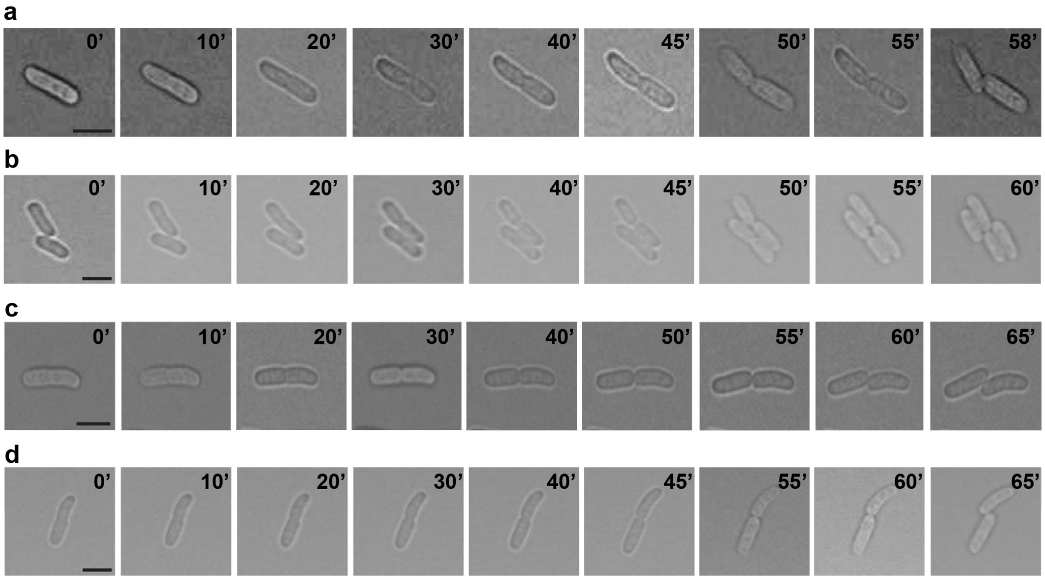

Cell generation times of strains.

Cells were imaged under bright field illumination every 1-3 minutes. All strains had comparable cell cycle times. (**a**), WT MG1655, average cell cycle time 59 ± 3 minutes (*n* = 25). (**b**), BS001 (FtsZ-GFP) showed an average cell cycle time of 61 ± 4 minutes (*n* = 36). (**c**), BS007 (GFP-FtsI) showed an average cell cycle time of 60 ± 2 minutes (*n* = 11). (**d**), EC1213 (GFP-FtsN) showed an average cell cycle time of 65 ± 7 minutes (n = 21). Scale bars = 2 μm.

Supplementary Figure 7

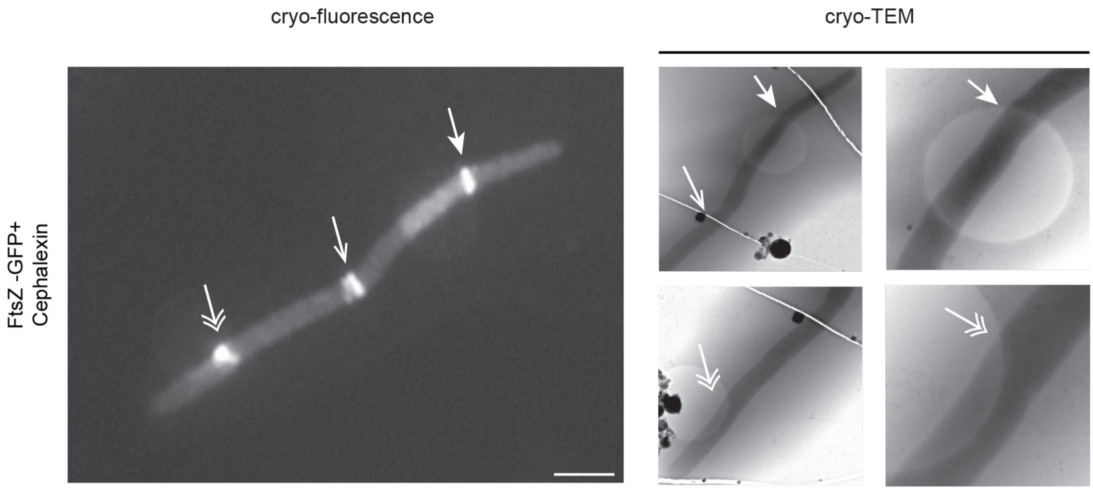

FtsZ-GFP is not generating enough force to constrict the membrane in Cephalexin treated cells.

Cells expressing FtsZ-GFP was treated with Cephalexin (15 μm/ml) for ~ 30-45 minutes and subjected to cryo-CLEM. The cryo-fluorescence image above depicts a representative cells with multiple FtsZ-GFP rings. The 3 arrows (double headed, single headed thin and single headed thick, respectively) points to possible division sites as defined by the accumulated GFP signal, and the corresponding arrows point to the same locations in the cryo-EM images. No invaginations were detected under the FtsZ-GFP fluorescence, which further supports the conclusion that FtsZ is not generating enough contractile force to constrict the membranes. In total were 27 FtsZ-GFP rings in 6 cells examined. Scale bar = 2 μm.

Supplementary table 1

Name	Strain	Plasmid backbone	Fluorescent protein expressed	Antibiotic marker	Inducer	Source
BS001	MG1655	--	FtsZ-GFP	Amp^R	2.5 µM IPTG	[8]
BS003	MG1655	--	FtsA-GFP	Amp^R	100 µM IPTG	[8]
BS007	MG1655	--	GFP-FtsI	Amp^R	5 µM IPTG	[8]
EC1213	MG1655	--	GFP-FtsN	$Kana^R$	5 µM IPTG	[5]
pEG4	--	pBAD33	FtsZ-mCherry	$Chlora^R$	0.2 % (w/v) Arabonise	[9]
pST1	--	pRha67	ZipA-mCherry	$Kana^R$	5 mM Rhamnose	[5]

Strains and plasmids.

Strains and plasmid used in this work.

Supplementary references:

1 Dubochet, J. *et al.* Cryo-electron microscopy of vitrified specimens. *Q. Rev. Biophys.* **21**, 129-228 (1988).

2 Chang, Y. W. *et al.* Correlated cryogenic photoactivated localization microscopy and cryo-electron tomography. *Nat. Methods* **11**, 737-739 (2014).

3 Schwartz, C. L., Sarbash, V. I., Ataullakhanov, F. I., McIntosh, J. R. & Nicastro, D. Cryo-fluorescence microscopy facilitates correlations between light and cryo-electron microscopy and reduces the rate of photobleaching. *J. Microscopy* **227**, 98-109 (2007).

4 Sun, Q. & Margolin, W. FtsZ dynamics during the division cycle of live *Escherichia coli* cells. *J. Bacteriol.* **180**, 2050-2056 (1998).

5 Söderström, B. *et al.* Coordinated disassembly of the divisome complex in *Escherichia coli. Mol. Microbiol.* **101**, 425-438 (2016).

6 Gerding, M. A. *et al.* Self-enhanced accumulation of FtsN at Division Sites and Roles for Other Proteins with a SPOR domain (DamX, DedD, and RlpA) in *Escherichia coli* cell constriction. *J. Bacteriol.* **191**, 7383-7401 (2009).

7 Busiek, K. K. & Margolin, W. A role for FtsA in SPOR-independent localization of the essential *Escherichia coli* cell division protein FtsN. *Mol. Microbiol.* **92**, 1212-1226 (2014).

8 Söderström, B. *et al.* Disassembly of the divisome in *Escherichia coli*: evidence that FtsZ dissociates before compartmentalization. *Mol. Microbiol.* **92**, 1-9 (2014).

9 Galli, E. & Gerdes, K. Spatial resolution of two bacterial cell division proteins: ZapA recruits ZapB to the inner face of the Z-ring. *Mol. Microbiol.* **76**, 1514-1526 (2010).
